# Role of Glyoxalase in Astrocytes’ Supportive Function Under Hyperglycemic Conditions: Aminoguanidine and Kir4.1 Channel Recovery

**DOI:** 10.3390/brainsci15101075

**Published:** 2025-10-03

**Authors:** Jadier Colón-Vázquez, Nathaly M. Rosado-Rivera, Joshua J. Navedo-Jackson, Arelys A. Angueira-Laureano, Yanitza Hernandez-Santiago, Geronimo Maldonado-Martinez, Miguel P. Méndez-González, Misty J. Eaton, Serguei N. Skatchkov, David E. Rivera-Aponte

**Affiliations:** 1 Department of Biochemistry, School of Medicine, Universidad Central del Caribe, Bayamon, PR 00956, USA; 124jnavedo@uccaribe.edu (J.J.N.-J.); 424yhernandez@uccaribe.edu (Y.H.-S.); misty.eaton@uccaribe.edu (M.J.E.); serguei.skatchkov@uccaribe.edu (S.N.S.); 2 Department of Biochemistry and Molecular Biology, Universidad Complutense de Madrid, 28040 Madrid, Spain; nathaly.rosado@upr.edu; 3 Department of Medicine, School of Medicine, University of Puerto Rico, Medical Science Campus, San Juan, PR 0093, USA; arelys.angueira@upr.edu; 4 Biology Department, University of Puerto Rico, Bayamon, PR 00956, USA; 5 Comprehensive Cancer Center, University of Puerto Rico, San Juan, PR 00921, USA; gmaldonado@cccupr.org; 6 Natural Sciences Department, University of Puerto Rico, Aguadilla, PR 00603, USA; miguel.mendez3@upr.edu; 7 Sciences and Technology, Antillean Adventist University, Mayagüez, PR 00681, USA; 8 Department of Physiology, School of Medicine, Universidad Central del Caribe, Bayamon, PR 00960, USA

**Keywords:** diabetes, astrocytes, glyoxalase system, aminoguanidine, Kir4.1 channels, potassium uptake, membrane potential

## Abstract

Background/Objectives: Diabetes mellitus is a metabolic disorder, and hyperglycemia results in abnormal brain function. Since glycolysis is the main energy pathway in glial cells, astrocytes possess a more developed glyoxalase (Glo) system than neurons and exhibit better survival. Glycolysis helps to protect glia from (i) dicarbonyl stress and (ii) formation of advanced glycation end products (AGEs). Since aminoguanidine (AG) is an inhibitor of AGE production, the purpose of this study was to determine the role of AG in crucial astrocytic proteins, such as Kir4.1, Glo1, and Glo2, in hyperglycemic conditions. Methods: We cultured astrocytes in normal (5 mM)- and high (25 mM)-glucose conditions. After two weeks, we seeded the cells in six-well plates, with 300,000 cells/well, and then treated them with 9 mM of AG for 24 h. Results: Expression of the glyoxalases Glo1 and Glo2, and of Kir4.1, is decreased in hyperglycemic conditions; however, treatment with AG recovers the expression of the Kir4.1 protein as well as the inward currents of hyperglycemic astrocytes. Conclusion: We demonstrated that regulation of the glyoxalase system via AG or another scavenger of carbonyl and aldehydes containing polyamine groups can contribute to the recovery of astrocyte function in diabetic patients.

## 1. Introduction

Diabetes mellitus disrupts glucose metabolism. Prolonged high blood sugar negatively affects the central nervous system (CNS) and leads to neuronal dysfunction, increasing vulnerability to injury. In the brain, high glucose can cause neurotoxicity, leading to abnormal CNS function and trauma [1]. People with diabetes are at a higher risk of stroke compared to people without diabetes, and high blood sugar during a stroke is linked to poor outcomes [2]. Additionally, increased glucose concentrations raise the levels of reactive oxygen species, leading to oxidative stress [3] and an increase in inflammatory cytokines [4], which reduces astrocyte functionality.

Astrocytes are the most abundant cells in the CNS cortex [5]. They support neurons and play a role beyond just structural support [6]. Astrocytes help regulate the extracellular environment by buffering ions (K^+^, H^+^) and neurotransmitters such as glutamate and GABA. They also form specialized synaptic connections with neurons, utilizing neuroactive substances like K^+^, H^+^, polyamines (PA), GABA, and glutamate [7,8] and forming tripartite [9] and tetrapartite synapses [10]. Glial cells have a 20–30 mV more hyperpolarized membrane potential than neurons. This potential provides the driving force needed for K^+^ and glutamate buffering [11,12,13] and PA transport [14,15]. Kir4.1 channels, expressed by the Kcnj10 gene, are widely found in glia [16, 17,18] and are the main pathway for K^+^ buffering. Their structure, which has two transmembrane segments and an extracellular pore loop, provides both ion selectivity and sensitivity to polyamines. Mutations or variations in the Kcnj10 gene compromise Kir4.1 function in glia [19,20]. This can lead to severe EAST/SeSAME [21,22] and Rett [7] syndromes, seizure susceptibility [12], and other disorders [14,23]. Kir4.1 channels consist of two transmembrane regions with one extracellular loop, containing sequences that contribute to ion-selective filtering for K^+^ and PA blockage [23]. These potassium channels are crucial for absorbing K^+^ released by neurons during action potential propagation [12,24,25,26]. Without these channels, glial cells struggle to remove glutamate from the extracellular space [10,11,12,24,25,27,28,29,30,31].

A single action potential is capable of raising extracellular K^+^ close to 1 mM, while neuronal discharge at a high frequency can increase it by several millimoles [28]. Ischemic conditions may lead to an elevation in extracellular potassium up to 45 mM in the hippocampus and 35 mM in the cortex [32]. Such an increase in extracellular K^+^ can compromise the normal function of the neurons. Healthy glial cells are located around the neuronal synapses and take up excess K^+^ ions from the synaptic cleft using transporters such as Na^+^/K^+^ ATPase- and K^+^-selective ion channels, predominantly inward rectifier Kir4.1 and tandem pore domain K^+^ channels such as TREK-1,2 and TASK-1 [7,11,24,25,29,30,33]. Using Kir4.1 channels, astrocytes take up excess K^+^ [6,12] and glutamate [11,25]. Using Cx43 gap junctions (GJs), astrocytes redistribute molecules [34] and K^+^ ions through the polyamine-dependent cell-to-cell network [15] to other astrocytes in the isopotential syncytium formed by Cx43 GJs [35]. GJs transfer K^+^ from zones of high concentrations of K^+^ to zones of low concentrations and to areas where the endfeet of the astrocytes enwrap the blood vessels [17,35].

In addition, the glial glutamate transporters, GLAST and GLT-1, work together with Kir4.1 [11,25], and can take up glutamate by coupling transport of Na^+^ and K^+^ down their concentration gradients—with inward movement of glutamate and sodium to the cytoplasm—with K and H^+^ out of the astrocyte [36,37]. This transport is electrogenic, meaning glial cells take up one glutamate with three Na+ and release one K^+^ and two H^+^, driven by electric and ionic gradients when the transporters are activated. There are two forms of regulation of Na^+^-dependent glutamate transport: (i) regulation of transporter expression or (ii) regulation of thermodynamic factors that can compromise the driving force for glutamate uptake, for example, Na^+^ gradients, K^+^ gradients, H^+^ gradients, and membrane potential [36,37]. In pathological cases, such as ischemia with dissipation of the ionic gradient, the transporter may run backwards, releasing glutamate back to neurons, thus triggering their death via glutamate toxicity [37]. Such reversal of the transporter is possible when glial cells are strongly depolarized [19,37].

Previously published studies showed that (i) astrocytes treated with siRNA against Kir4.1 [11], or (ii) astrocytes from seizure-susceptible DBA-2 mice [12], exhibit reduced Kir4.1 expression, decreased K-currents, impaired potassium and glutamate uptake [11,12], and disrupted synaptic function [12,28,38], similarly to astrocytes cultured in hyperglycemic conditions [26,39,40]. In addition, glutamate transport in male mice is more sensitive to the activator of GLT-1 than that in females [41]. It was also demonstrated that hyperglycemia downregulates Kir4.1 potassium channel expression via miR-205 [39]. Moreover, astrocytes from type 2 diabetic male mice have a depolarized membrane potential with impaired K^+^ uptake, while their neurons exhibit epilepsy-like hyperexcitability [40].

People with diabetes have increased susceptibility to developing epileptiform-like activity, cognitive impairment, and neurodegenerative diseases such as stroke, yet the mechanism of this increased susceptibility remains unknown [42,43]. Compromised astrocytes have a reduced ability to maintain extracellular homeostasis and can disrupt the normal function of neurons [44]. Important functions that, if disrupted, could result in neuron death include glutamate uptake and release, potassium buffering, free-radical scavenging, and the production of cytokines and nitric oxide [45].

Astrocytes possess a more developed glyoxalase system than neurons, which helps to protect them from dicarbonyl stress and advanced glycation end product (AGE) formation by proteins, nucleic acids, and lipids, induced by high levels of methylglyoxal (MG) [46]. Hyperglycemia increases the levels of AGEs and MG, which are extremely toxic to astrocytes. Therefore, keeping MG levels under control is crucial for the prevention of AGE formation [47]. When glucose levels are high, MG increases due to impairments in the glyoxalase system, promoting the formation of AGE and affecting the glutamate uptake function in astrocytes [48]. The glyoxalase system is important for detoxifying MG and, therefore, for keeping MG concentrations under control, and preventing AGE formation [47]. The glyoxalase system consists of Glyoxalase 1 (Glo1) and Glyoxalase 2 (Glo2). This system detoxifies MG via hemithioacetal; it is then converted to S-D-lactoyglutathione and hydrolyzed by Glo2 into D-lactate. Glo1 works as the rate-limiting step in the MG detoxification process, and it is believed that Glo1 maintains the intracellular concentration of MG at a lower level.

Aminoguanidine (AG), an inhibitor of several oxidation reactions conducted by enzymes such as diamine oxidases and polyamine oxidases [49], is known for its ability to impede the formation of AGEs by trapping reactive carbonyls formed during the Maillard reaction, especially Amadori intermediates. AG can also interact with dicarbonyl compounds like methylglyoxal (MG), glyoxal, and 3-deoxyglucosone [49,50]. It can prevent the formation of glycoxidation and lipoxidation products and interrupt vicious oxidative damage cycles [49,50].

Previous studies show that astrocytes treated with 4 mM MG decrease glutamate uptake [50]; however, treating astrocytes with AG after exposure to MG leads to a decrease in MG levels [50]. It could be suggested that since glutamate uptake is not functional without Kir4.1 channels [11], the MG-Glo-system regulates glutamate uptake, which in turn depends on AG [50]. Therefore, the recovery of Kir4.1 channels in hyperglycemic conditions would be sensitive to AG as well. The purpose of this study was to determine the role of AG in astrocytes cultured in hyperglycemic conditions. Our hypothesis is that using AG will increase Kir4.1 protein expression and the function of Kir4.1 potassium channels, and thereby, the astrocytic Kir4.1 channels will increase whole-cell current contribution.

## 2. Materials and Methods

### 2.1. Primary Cell Culture

For astrocyte culture, the neocortex of 1–2-day-old Sprague Dawley rats was used following the protocol approved by the Institutional Animal Care and Use Committee (protocol #058-2023-13-000) [12]. To summarize, after decapitation the brains were removed, followed by stripping the meninges to minimize fibroblast contamination. The forebrain cortices were dissociated using the stomacher blender method. Cell suspension was filtered via gravity through a #60 sieve and then through a #100 sieve. Cells were then centrifuged and divided into 2 groups; these groups were plated in uncoated 75 cm^2^ flasks at a density of 300,000 cells/cm^2^. The first group was plated in high-glucose Dulbecco’s Modified Eagle Medium (DMEM) containing 25 mM glucose, 2 mM glutamine, 1 mM pyruvate, 10% fetal bovine serum, and 100 iU/mL penicillin/100 μg/mL streptomycin. The second group was plated in normal-glucose DMEM, where the glucose concentration was 5 mM. The medium was replaced with the appropriate fresh culture medium every 4 days. At confluence (about 12 days), the mixed glial cultures were treated with 50−75 mM L-Leucine methyl ester (LME) (pH 7.4) for 10–20 min to eliminate microglia. Cultures were then allowed to recover for at least one day in growth medium prior to experimentation. Astrocytes were dissociated by trypsinization and reseeded onto appropriate plates for the experiments.

### 2.2. Aminoguanidine Treatment

Dose- and time-course analyses were performed in order to determine the best conditions for the treatment of cells with AG. Cells were treated with aminoguanidine at 9 mM for 24 h. After treatment, cells were collected for Western blot or seeded on coverslips for electrophysiology.

### 2.3. SDS-PAGE and Western Blot Analysis

The lysis buffer contained phenylmethylsulfonyl fluoride (PMSF), protease inhibitor, and Radioimmunoprecipitation Assay (RIPA) buffer, the latter of which contained Tris HCl 1.5 M pH 8.8, 1% Triton X-100, 150 mM NaCl, and 0.1% SDS. The lysates were mixed with Urea sample buffer (plus β-Mercaptoethanol), boiled, and spun briefly to pellet debris. In most cases, samples were immediately run on 10% SDS-polyacrylamide gels, and some samples without the Urea sample buffer were stored at −80 °C. The protein concentration of cell homogenates was determined via a Bradford assay (Bio-Rad, Hercules, CA, USA), followed by addition of an appropriate volume of Urea sample buffer (62 mM Tris/HCl pH 6.8, 4% SDS, 8 M Urea, 20 mM EDTA, 5% β-Mercaptoethanol, 0.015% Bromophenol Blue) to a final concentration of 15 μg protein/μL, and incubation in a water bath at 100 °C for 10 min.

Western blotting was performed as previously described [39], using antibodies prepared in 5% Bovine Serum Albumin (BSA) Kir4.1 (1:2000, Cat #:APC-035-GP Alomone, Jerusalem, Israel), Glyoxalase 1 (1:1000, Cat # MA1-13029 Invitrogen, Waltham, MA, USA), and Glyoxalase 2 (1:1000, Cat # PA5-93097 Invitrogen, MA, USA). Final detection was performed with an enhanced chemiluminescence methodology (SuperSignal^®^ West Dura Extended Duration Substrate; Pierce, Rockford, IL, USA) as described by the manufacturer, and the intensity of the signal was measured in a gel documentation system (Versa Doc Model 1000, Bio Rad). In all cases, the chemiluminescence intensity was corrected to address minor differences in protein content after India ink-stained densitometry analysis of the membranes.

### 2.4. Electrophysiology

Whole-cell recordings were performed using a single-electrode patch-clamp. After break-in, we measured resting membrane potential in the current clamp and membrane currents under voltage-clamp conditions. Perfusing micropipettes, with a tip diameter of 30–50 μm, were positioned using hydraulic micromanipulators to enable controlled solution exchange and to allow rapid application of the solutions. Membrane currents were collected using the single-electrode whole-cell patch-clamp technique, following methods described previously [12,24]. Voltage-clamp recordings and micropipette positioning were performed with two Narishige hydraulic micromanipulators (MMW-203, Setagaya, Tokyo, Japan). The patch-clamp electrodes were made from borosilicate glass capillaries (1B150F-4, World Precision Instruments, Sarasota, FL, USA) using a two-step process with a Sutter P-97 puller (Sutter Instruments Corp., Novato, CA, USA). The recording microelectrodes were filled with intracellular solution (ICS) and had a resistance of 4–6 MΩ. The ICS contained specific ion concentrations, while the extracellular solutions were made with standard ionic compositions optimized for astrocyte recordings, maintaining physiological osmolarity and pH. The ICS contained 120 mM K-gluconate, 10 mM KCl, 1 mM MgCl_2_, 1 mM CaCl_2_, 10 mM EGTA, 10 mM HEPES, and 0.3 mM spermine tetrahydrochloride, and the pH was adjusted to 7.2 with KOH/HCl. After achieving whole-cell mode, access resistance was 10–15 MΩ, compensated by at least 75%. The extracellular solution (ECS) included 145 mM NaCl, 2.5 mM CaCl_2_, 2 mM MgCl_2_, and 10 mM HEPES. KCl concentrations varied from 1.0 to 10 mM, with NaCl substituted to maintain osmolarity. The control solution contained 3 mM KCl. Signals were amplified using an Axon Multiclamp-700B amplifier and a CV-203BU headstage. They were filtered at over 1 kHz, digitized at 5 kHz with a DigiData 1322A interface (Axon Instruments, Molecular Probes, Sunnyvale, CA, USA), and analyzed with the pClamp 9 software package (Axon Instruments, Union City, CA, USA).

### 2.5. Statistical Analysis

Quantification and normalization of Western blot signals were performed with Image Lab-BioRad. Western blot quantification was analyzed with Student’s t-test, and experiments performed with multiple groups were analyzed with one-way ANOVA followed by Tukey’s multiple comparison test. A value of *p* < 0.05 was considered significant. The collected Western blot data files were analyzed using GraphPad Prism v10.4.1 (GraphPad, San Diego, CA, USA).

For the barium-sensitive currents, we subtracted the currents obtained in the presence of barium from the total whole-cell currents shown. The complete analysis was performed using a two-way ANOVA followed by Tukey’s multiple comparison test. Data files were analyzed using GraphPad Prism (GraphPad, San Diego, CA, USA).

## 3. Results

Protein expression and functional consequences of hyperglycemia: role of AG in Glo1, Glo2, and Kir4.1 protein expression, and in Kir4.1 channel function.

### 3.1. Protein Expression of Glyoxalase 1 and 2 Is Downregulated in High-Glucose Conditions When Compared to Control

**Figure 1:**
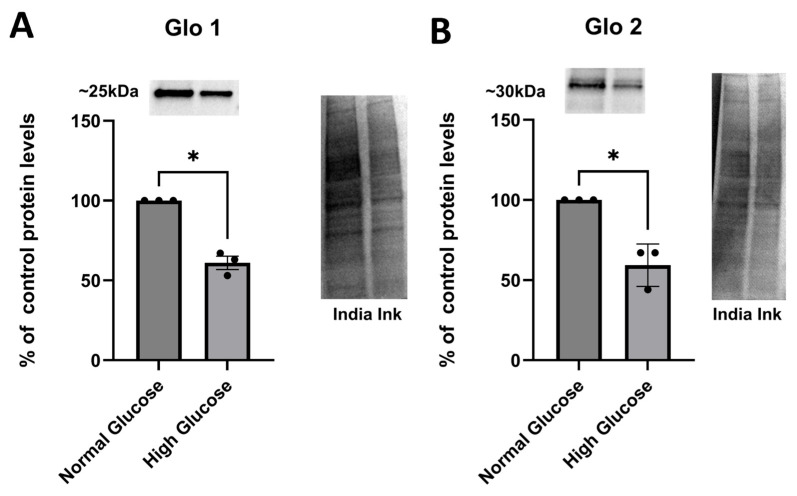
Western blot analysis of Glo1 and Glo2 protein levels in normal and hyperglycemic conditions. Astrocytes were plated in normal (300,000 cells/well) (5 mM)- and high (25 mM)-glucose conditions (300,000 cells/well) for 2 weeks. (**A**). Glo 1 protein expression was significantly reduced in astrocytes cultured in high-glucose conditions when compared to the control (−39.00% in relation to control ± 4.163 SEM, *n* = 3). (**B**). Glo2 protein expression was significantly reduced in astrocytes cultured in high-glucose conditions when compared to the control (−40.67% in relation to control ± 7.667 SEM, *n* = 3). Quantification was normalized utilizing India ink (* *p* < 0.05).

We hypothesized that Glo1 and Glo2 will be downregulated in hyperglycemic conditions. To test our hypothesis, we cultured astrocytes in normal-glucose conditions (5 mM) and high-glucose conditions (25 mM). After a two-week growth period, we treated them with LME to eliminate microglia and ensure a pure culture of just astrocytes. A few days after treatment, cells were collected and lysed for protein extraction. Protein quantification was performed with the Bradford assay, and protein levels were determined via Western blot. Our results show that astrocytes cultured in high-glucose conditions have lower expression of Glyoxalase 1 (Figure 1A) and Glyoxalase 2 (Figure 1B) than those cultured in normal conditions.

### 3.2. Kir4.1 Protein Expression Is Downregulated in High-Glucose Conditions When Compared to Control: Role of AG in Recovering Kir4.1 Expression

It was demonstrated that Kir4.1 protein expression is decreased in astrocytes cultured in hyperglycemic conditions [39]. We now examined if Kir4.1 channel protein expression being decreased in hyperglycemic conditions can be prevented. As suggested above, we hypothesized that aminoguanidine (AG) would prevent Kir4.1 downregulation in hyperglycemic conditions. To further evaluate the effect of AG, we cultured astrocytes in control DMEM with 5 mM glucose and high DMEM (25 mM). After two weeks, we plated the cells in a six-well plate containing 300,000 cells/well, which we treated with 9 mM of AG for 24 h. We determined protein levels using a Western blot.

**Figure 2:**
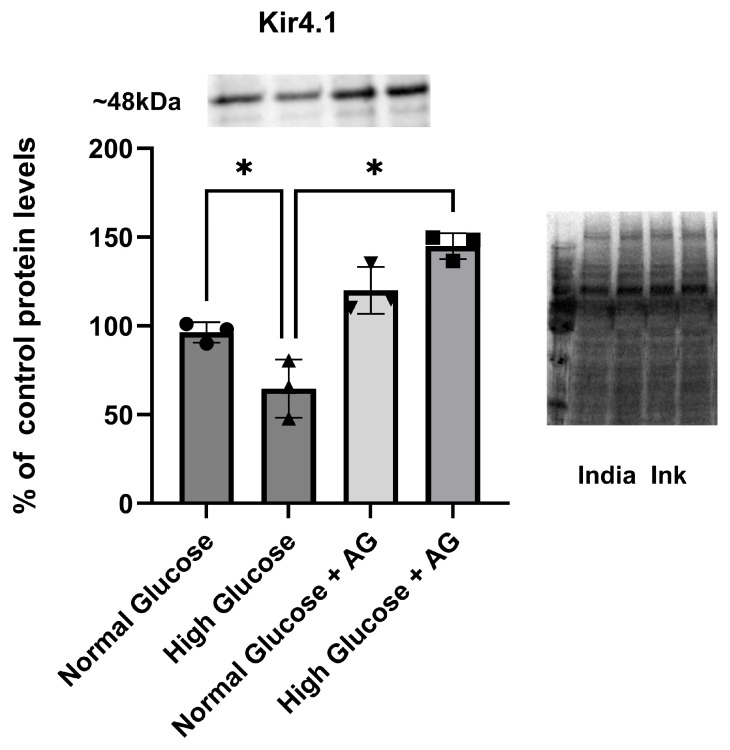
Western blot analysis of Kir4.1 protein levels in normal and hyperglycemic conditions: Effect of aminoguanidine (AG). Astrocytes were grown in normal conditions (300,000 cells/well), 5 mM (normal glucose), and in hyperglycemic conditions (300,000 cells/well), 25 mM (high glucose). After 2 weeks, astrocytes were treated with 9 mM of AG for 24 h (normal glucose + AG; high glucose + AG, 300,000 cells/wells). Kir4.1 protein expression is downregulated in high-glucose conditions; however, when treated with AG for 24 h, there is significant recovery in the protein expression of Kir4.1 in astrocytes, specifically those cultured in high-glucose conditions with AG when compared to astrocytes cultures grown without AG (*p* = 0.0001, 95% C.I [−109.3 to −51.01]). Quantification was normalized utilizing India ink (* *p* < 0.05).

Astrocytes grown in normal conditions, 5 mM (normal glucose), and in hyperglycemic conditions, 25 mM (high glucose), contain different amounts of Kir4.1 protein. Hyperglycemic astrocytes showed a reduction in Kir4.1 protein of about 30%; however, after treatment with 9 mM of AG for 24 h (normal glucose + AG; high glucose + AG) the astrocytes showed an increase in Kir4.1 protein expression compared with the control (Figure 2). Significant recovery of Kir4.1 protein of about two times was observed in astrocytes, specifically those cultured in high-glucose conditions and treated with AG, compared to astrocytes grown without AG (*p* = 0.0001, 95% C.I [−109.3 to −51.01]). Quantification was normalized utilizing India ink (Figure 2, * *p* < 0.05). Our results show that even astrocytes cultured in normal-glucose conditions (5 mM) exhibited increased Kir4.1 protein expression when treated with 9 mM of AG, compared to untreated astrocytes (Figure 2). To further clarify the effects of growing Kir4.1 expression induced by AG, we conducted two different sets of experiments to figure out the functional properties of Kir 4.1 channels, including (i) membrane potentials (Section 3.3) and (ii) membrane currents (Section 3.3).

### 3.3. Barium-Sensitive Kir4.1 Current Is Increased in Astrocytes Grown in High-Glucose Conditions and Treated with Aminoguanidine

**Figure 3:**
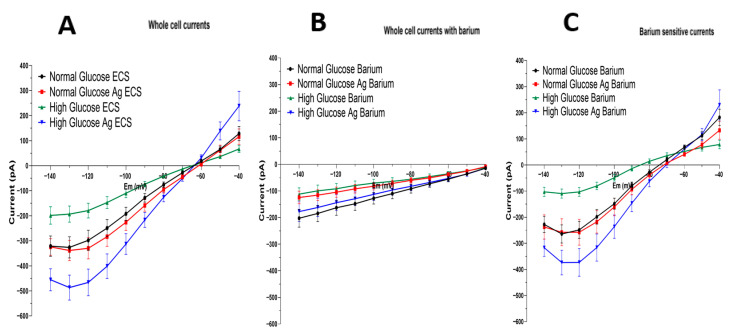
Astrocytic Kir4.1 currents in normal and hyperglycemic conditions: effect of aminoguanidine (AG). Astrocytes were cultured in normal (5 mM) or high (25 mM) glucose for two weeks and plated on coverslips, followed by treatment with 9 mM of AG. Astrocytes were clamped at the holding potential (Eh), which was equal to the resting membrane potential (Em) in 3 mM [K^+^]_o_. Eh = Em gives zero current. I/V-curves are shown in response to a voltage step protocol: 10 mV increments from −140 mV to −40 mV, at 70 mV below and 100 mV above the holding potential (Eh). The left panel represents whole-cell currents, the middle panel shows residual-cell currents after barium treatments, and the right panel represents pure Kir-channel currents. (**A**). Whole-cell currents recorded from astrocytes grown in normal glucose (*n* = 24), normal glucose treated with AG (*n* = 32), high glucose (*n* = 25), and high glucose treated with AG (*n* = 27) in response to a voltage-clamp protocol. (**B**). Whole-cell currents recorded from astrocytes grown in normal glucose (*n* = 26), normal glucose with AG treatment (*n* = 28), high glucose (*n* = 25), and high glucose with AG treatment (*n* = 27) in response to a voltage-clamp protocol in the presence of 100 μM Ba^2+^ (a blocker of Kir channels). (**C**). Barium-sensitive currents from astrocytes grown in normal glucose (*n* = 21), normal glucose with AG treatment (*n* = 22), high glucose (*n* = 21), and high glucose with AG treatment (*n* = 24). The graph shows the subtraction of currents obtained in the presence of barium (**B**) from total whole-cell currents shown in (**A**). Barium-sensitive currents reflect the contribution of Kir channels to the whole-cell currents. There was a significant difference between inward currents of high-glucose astrocytes treated with AG and high-glucose astrocytes without treatment (*p* = 0.0001, 95% C.I [−234.1 to −31.19]). The green curve in the right panel shows a decrease in Kir4.1 currents in hyperglycemic conditions. However, aminoguanidine restores Kir4.1 currents (blue curve) (data was analyzed using a two-way ANOVA followed by a Tukey test).

Astrocytes were clamped at holding potential (Eh) equal to the resting membrane potential (Em) in 3 mM [K^+^]_o_. Therefore, the difference between Eh and Em gives zero voltage gradient at the membranes of astrocytes and, consequently, zero current, which prevents both the accumulation and release of potassium and other ions from the cells. Barium in micromolar concentration is a blocker of Kir4.1 currents; however, in a millimolar concentration it may also partially depress 2P channel currents [33]; therefore, we used low-dose barium. We evaluated the effects AG had on inward-rectifying currents when they were blocked by a low dose of barium, a specific Kir-channel blocker. The current I/V-curves of the astrocytes are shown in Figure 3, which were obtained via a voltage step protocol: voltage increased in 10 mV increments from −140 mV to −40 mV, at 70 mV below and 100 mV above the holding potential. Figure 3A shows whole-cell currents recorded from astrocytes grown in (i) normal glucose (*n* = 24), (ii) normal glucose treated with AG (*n* = 32), (iii) high glucose (*n* = 25), and (iv) high glucose treated with AG (*n* = 27). We observed that astrocytes cultured in high-glucose conditions have significantly smaller cell currents when compared to the controls (Figure 3A). Hyperglycemia results in strongly diminished cell currents.

Figure 3B shows the effect of barium (Ba^2+^), the Kir4.1 channel blocker. Whole-cell currents recorded from astrocytes grown in normal glucose (*n* = 26), normal glucose with AG treatment (*n* = 28), high glucose (*n* = 25), and high glucose with AG treatment (*n* = 27) in response to a voltage-clamp protocol in the presence of 100 μM Ba^2+^. Barium in such a low dose blocked the Kir4.1 channels but not the TASK or TREK channels. Indeed, the currents lost their typical Kir-channel rectification characteristics, became linear (typical of 2P channels), and displayed small leak-like currents (Figure 3B).

Figure 3C shows the subtracted whole-cell currents (those without barium minus those with barium). Therefore, the barium-sensitive currents represent Kir4.1 channel currents from astrocytes grown in normal glucose (*n* = 21), normal glucose with AG treatment (*n* = 22), high glucose (*n* = 21), and high glucose with AG treatment (*n* = 24). There was a significant difference between inward currents of astrocytes grown in high-glucose-treated with AG and those of high-glucose astrocytes without treatment (*p* = 0.0001, 95% C.I {−234.1 to −31.19}). The green curve (Figure 3C) shows a decrease in Kir4.1 currents in hyperglycemic conditions. However, aminoguanidine restores Kir4.1 currents (Figure 3C, blue curve). Interestingly, AG has little or no effect on Kir4.1 currents of astrocytes grown in normal conditions (Figure 3C, red curve versus black curve), while AG has a dramatic effect on astrocytes grown in high-glucose conditions (green vs. blue curves). Namely, the inward potassium currents from hyperglycemic astrocytes were increased by AG treatment more than four times. Such a recovery of Kir4.1 channels suggests that AG and other diamines and polyamines represent promising molecular tools for restoring downregulated Kir4.1 channels in mechanical or chemical brain trauma, ischemia, etc.

## 4. Discussion

Previous studies show that high levels of glucose can cause alteration to the glycolytic pathway, resulting in elevated levels of toxic methylglyoxal (MG) in the system. With these elevated levels, the production of AGEs increases, causing inflammation, cell death, insulin resistance, and chronic diseases. Two key enzymes in MG detoxification, Glo1 and Glo2, work as a system that detoxifies the MG levels in the body into a less harmful product. Glo1 starts the process by combining MG with glutathione (GSH) to form S-D-lactoylglutathione, and Glo2 hydrolyzes S-D-lactoylglutathione, producing D-lactate and GSH. These enzymes are highly expressed in astrocytes, which helps astrocytes carry out their neuroprotective functions, including controlling MG levels and decreasing the creation of AGE due to MG. One of the key factors that increases MG is high blood glucose levels, and diabetic individuals are known to have elevated MG in their systems [46,47]. Excessive glucose metabolism leads to an increase in the production of MG [48]. With the glyoxalase system impaired, there is no direct detoxification against MG, causing an increase in AGEs and a decrease in astrocyte function and viability [50].

Hansen’s group demonstrated that elevated concentrations of MG in the system disrupt astrocytes’ glutamate uptake functionality; if these functions are altered, it will result in neurotoxicity. Intriguingly, while MG is elevated, the protein expression of glutamate transporters remains unchanged, meaning that the reduction in glutamate clearance is due not to the downregulation of glutamate transporters, but to other mechanisms. These consequences can be linked to the decreased levels of the Kir4.1 potassium channel [11]; other studies have demonstrated that increasing the level of MG causes a decrease in the expression of Kir4.1. It was demonstrated that hyperglycemia decreased the Kir4.1 gene and protein expression and decreased the functionality of membrane potential [20,39,40]. We have also shown that hyperglycemia decreases astrocytic function, such as glutamate uptake and potassium buffering, due to a reduction in the function and expression of the Kir4.1 potassium channel; taking these findings together with the findings of Hansen’s group, we are able to correlate the reduction in the glutamate clearance of astrocytes with the reduction in the Kir4.1 expression due to hyperglycemic conditions and MG levels [12,20,39,40].

Our findings show that hyperglycemic conditions cause a decrease in the protein expression of Glo1 and Glo2, as well as Kir4.1. With these critical players downregulated, we can expect elevated levels of MG, which disrupts astrocytes’ glutamate uptake, potassium buffering, and neuroprotective function, thereby increasing neurotoxicity. Having observed these results, we proceeded to utilize AG, which is known for its ability to impede the formation of AGEs and interact with MG, preventing glycoxidation by limiting the cycle of oxidative damage. AG protects against the formation of toxic radicals released during oxidation and improves early functional recovery after trauma [51]. Intriguingly, PA treatment repairs retinal neurons after trauma [52], and when PAs are combined with AG, it accelerates recovery after nerve injuries and promotes structural and functional recovery in the adult mammalian brain after trauma [51,53,54]. Moreover, PAs facilitate astrocyte survival when taken up [55]. Since AG contains several amino groups similar to Pas, treatment with exogeneous Pas has been shown to enhance neuronal survival [52]. AG, in combination with PAs, accelerates the early phases of motor function recovery [56]. It also enhances the rate of axon regeneration following trauma to the optic nerve [52] and facial nerve injury in adult rats [51], and after sciatic nerve lesions [57]. Polyamines (PAs) play a crucial role in various processes, including (i) central nervous system (CNS) diseases [7,58,59,60,61,62], (ii) synaptogenesis [63,64], (iii) the survival of astrocytes, and (iv) gliogenesis [55]. They aid in the regeneration of retinal neurons after injury and trauma [52], support longevity [65] and cardioprotection [66], improve memory, and initiate autophagy [58,67]. Recent research has shown that fasting therapy can lead to an accumulation of spermidine (SPD), which triggers autophagy and ultimately promotes increased longevity [67]. In studying diabetes, it is important to understand the role of both glial and neuronal cells, along with their channels and transporters [68], and how they are affected by AG and PAs. Since polyamines are taken up by astrocytes [55] it can be expected that AG will also be accumulated in these glial cells, which represents a promising avenue for future research.

Astrocytes cultured in high-glucose conditions lacked essential K-currents, and with AG treatment, the cells expressed an increase in Kir4.1 protein content and functional Kir4.1 currents. This leads us to believe that high-glucose conditions may increase AGE and MG expressions, causing a disruption to the membranes of astrocytes and thus preventing them from maintaining proper function, such as K-buffering and supporting glutamate transport, yet the precise mechanism of this disruption remains unknown.

Furthermore, after observing the increase in Kir4.1 protein expression in astrocytes cultured in high-glucose conditions when AG is present, we specifically evaluated the inward-rectifying channels by using barium, a specific Kir blocker (Figure 3). We observed that astrocytes cultured in high-glucose conditions have lower cell currents when compared to the controls (Figure 3A). Administration of barium disrupts Kir4.1 channel function and results in a lower current, which is almost completely diminished (Figure 3B). We observed that astrocytes cultured in high-glucose conditions exhibited an increase in currents and barium-sensitive currents when compared to untreated astrocytes, and we were surprised to see that these astrocytes also increased currents compared to the normal-condition astrocytes. This leads us to theorize that Kir4.1 channels are potentially influenced by MG and AGE levels, with higher levels causing disruption in the membrane currents and their activity. Controlling these levels utilizing AG may be the key to protecting astrocyte function, which is necessary for maintaining homeostasis for neurons and reducing neuronal death caused by increased levels of glutamate and potassium in the synaptic cleft.

## 5. Conclusions

In conclusion, our results show that hyperglycemia causes (i) a decrease in Glo1 and Glo2 enzyme content, (ii) decreased Kir4.1 protein expression levels, and (iii) downregulation of the functional Kir4.1 channel current. However, AG treatment recovers the expression and function of Kir4.1 channels in astrocytes. Further experiments on the glyoxalase system (MG, AGES, AG, GLO1, and 2) during hyperglycemic conditions can provide new insight into the mechanism of combatting hyperglycemia with AG, and with other diamines and polyamines, and into increasing the health status of the CNS.

## Data Availability

The original contributions presented in this study are included in the article. Further inquiries can be directed to the corresponding author.

## Abbreviations

The following abbreviations are used in this manuscript:

AG	Aminoguanidine
PAs	Polyamines
AGE	Advance glycation end product
CNS	Central nervous system
Em, Eh	Membrane and holding potentials, respectively
GABA	Gamma amino butiric acid (a gliotransmitter and neurotransmitter)
GLAST, GLT1	Glutamate transporters
Glo 1, 2	Glyoxalases 1 and 2
GSH	Glutathione
Kcnj10	Gene-encoding subunit protein of Kir4.1 channels
Kir4.1	Potassium inward-rectifying channels expressed in glia
MG	Methylglyoxal
TASK-1	Two-pore domain acid-sensitive potassium channel, gene KCNK3
TREK-1	Two-pore domain acid-sensitive potassium channel, gene KCNK2
TREK-2	Two-pore domain acid-sensitive potassium channel, gene KCNK10
Cx 43 GJs	Connexin-43 gap junctions (pores between astrocytes in the glial syncytium)
